# Microstructural Engineering of Ferroelectric and Electromechanical Properties in 0.65KBT-0.35BCZT Ceramics

**DOI:** 10.3390/ma18030623

**Published:** 2025-01-29

**Authors:** Mohammed N. Al-Aaraji, Bing Wang, Antonio Feteira, David A. Hall

**Affiliations:** 1 Department of Ceramic Engineering and Building Materials, College of Materials Engineering, University of Babylon, Babylon 51002, Iraq; 2 Department of Materials, University of Manchester, Manchester M13 9PL, UK; 3 College of Civil and Transportation Engineering, Shenzhen University, Shenzhen 518060, China; kingsky1013@126.com; 4 Institute for Advanced Study, Shenzhen University, Shenzhen 518060, China; 5 Materials and Engineering Research Institute, Sheffield Hallam University, Sheffield S1 1WB, UK; a.feteira@shu.ac.uk

**Keywords:** ceramics, ferroelectric, solid-state reaction, quenching, microstructure, core-shell, electrostriction

## Abstract

The influence of processing procedures and microstructural features on the functional properties of relaxor ferroelectric ceramics are of fundamental interest and directly relevant to their applications in dielectric capacitors and electromechanical sensors/actuators. In the present work, solid solutions of 0.65(K_0.5_Bi_0.5_)TiO_3_-0.35(Ba_0.94_Ca_0.06_)(Ti_0.93_Zr_0.07_)O_3_ (0.65KBT-0.35BCZT) were processed by solid-state reaction using two different procedures, distinguished in terms of mixed or separate calcination of the KBT and BCZT components and leading to homogeneous or core-shell-type relaxor ferroelectric ceramics, respectively. Systematic research was conducted on the impact of the processing techniques and air-quenching procedures on the structure and ferroelectric and electromechanical properties. Higher remanent polarization of the separately calcined materials was ascribed to the ferroelectric nature of the core regions, along with the non-ergodic relaxor ferroelectric response in the shell, which was enhanced by the quenching process. It was also demonstrated that the thermal depolarization temperature increased significantly after quenching, from ~100 to ~160 °C for the separately calcined ceramic, and from ~50 to ~130 °C for the mixed material; moreover, these effects are linked to notable improvements in the ferroelectric tetragonal phase content by air-quenching.

## 1. Introduction

Electromechanical coupling in piezoceramics is employed in sensors, ultrasonic transducers, and actuator technologies [1,2,3]. For many years, lead-based, perovskite-structured ferroelectric ceramics, such as lead zirconate titanate (PZT), have been considered the most suitable in terms of their piezoelectric performance and temperature stability. However, the presence of toxic lead has been recognized as a significant issue since the implementation of the RoHS/WEEE regulations has come into effect [4,5,6]. Consequently, many researchers have focused on the development of lead-free piezoelectric ceramics that have a performance comparable with that of lead-containing ceramics, due to the global demand for lead-free materials to safeguard the environment [7,8]. The most promising prospective lead-free candidate piezoceramic materials are often based on BaTiO_3_ (BT), (K,Na)NbO_3_ (KNN), and Bi_0.5_Na_0.5_TiO_3_ (BNT) in a solid solution with other perovskite compounds [9,10,11]. For applications in electromechanical actuators, BNT-based materials, for instance, have been shown to exhibit some of the largest electrostrain responses, achieving up to 0.45 % at 8 kV/mm [12]. These materials are typically characterized by the presence of polar nanoregions (PNRs), resulting in a pseudo-cubic structure and relaxor ferroelectric behavior, as observed in many bismuth-based perovskites [13,14]. The dynamic behavior of such PNRs under an applied electric field induces electrostrain, which can be classified as either a reversible (ergodic) or irreversible (non-ergodic) response [12,15,16,17]. However, there are some remaining issues that hinder the widespread exploitation of such materials. These include a relatively low depolarization temperature, significant hysteresis (>50%) in the strain–electric field relationship, and the need for a high electric field to initiate the relaxor to normal ferroelectric phase transformation [18,19].

Several approaches have been reported to overcome these drawbacks. Some of these involve the addition of other components to induce different types of chemical heterogeneity in the form of composite materials [20,21] or core-shell-type microstructure [17,22]. Furthermore, other perovskite components may be introduced to improve the reversibility of the phase switching behavior. For instance, a significant electrostrain with lower strain hysteresis and coercive field is produced when SrTiO_3_ (ST) is added to BNT at a concentration of between 22 and 28 mol% [23,24].

Other studies have focused on the microstructure–property relationships as a means to optimize the functional properties through the alteration of the processing parameters and techniques. For example, in the case of BNT-ST ceramics prepared using the conventional solid-state reaction method, several researchers have investigated the effects of milling time (2 to 24 h), calcination temperature (800 to 1100 °C), and sintering temperature (1050 to 1200 °C) [23,25,26]. Park et al. modified the process by separate pre-calcination of the BNT and ST components before sintering [27], while Krauss et al. added a stoichiometric mixture of SrCO_3_ and TiO_2_ to the pre-calcined BNT [28].

In a different approach, it has been reported that the quenching of bismuth-based perovskites from sufficiently high temperatures (~700 to 1100 °C) can lead to structural transformations and improve the thermal stability of ferroelectric characteristics [29,30,31]. For example, the depolarization temperature, *T_d_*, of BNT ceramics increased from 173 °C to 223 °C after air-quenching [32]. For MnO_2_-modified 0.75BiFeO_3_–0.25BaTiO_3_ ceramics, the use of quenching increased the remanent polarization, *P_r_*, from 0.06 to 0.31 C m^−2^ [33].

In previous publications [13,15], we investigated several approaches to enhance the ferroelectric characteristics of a related lead-free relaxor ferroelectric, K_0.5_Bi_0.5_TiO_3_ (KBT), with (Ba_0.94_Ca_0.06_)(Ti_0.93_Zr_0.07_)O_3_ (BCZT) being used as a second perovskite-structured component in the KBT-BCZT pseudo-binary solid solution. In that work, the components were pre-calcined separately and then mixed before sintering [13]. The resulting ceramics were characterized by a core-shell-type microstructure, which is a common feature in other Bi-based perovskite solid solutions [22,34,35,36]. Furthermore, air-quenching from 1000 °C yielded improved ferroelectric properties, higher tetragonal phase fraction (in the coexisting cubic and tetragonal phases) and enhanced tetragonal distortion in 0.65KBT-0.35BCZT ceramics [15]. The underlying mechanism(s) responsible for the effects of quenching on the crystal structure and ferroelectric properties in bismuth-based perovskite solid solutions are still controversial, but we have argued in previous articles that conventional slow-cooling processes may induce a certain degree of phase separation (on the nanoscale), driven by the increasing effects of thermodynamic immiscibility between some components of the solid solution upon cooling. The general disruption of ferroelectric ordering as a result of slow cooling after high temperature sintering or annealing is attributed to the development of local (atomic scale) variations in the chemical composition and ionic charge imbalance.

The present study was conducted to explore the effects of different solid-state processing procedures, in combination with air-quenching, on the structure and characteristics of 0.65KBT-0.35BCZT ceramics. This specific composition was selected on the basis of previous work, which demonstrated non-ergodic relaxor ferroelectric behavior at room temperature and a relatively high depolarization temperature in comparison with similar materials [13]. The potential for tailoring the ferroelectric properties through the structural and microstructural characteristics by suitable modification of the processing methods is investigated systematically. It is demonstrated that chemically homogeneous ceramics can be synthesized by a simplified one-step calcination method, while heterogeneous core-shell-type microstructures occur when a separate calcination method is employed.

## 2. Materials and Methods

Perovskite-structured 0.65(K_0.5_Bi_0.5_)TiO_3_-0.35Ba_0.94_Ca_0.06_(Ti_0.93_Zr_0.07_)O_3_ ceramics, abbreviated as 0.65KBT-0.35BCZT, were prepared using the conventional solid-state reaction method using 2 different strategies, denoted as *M* (mixed) or *S* (separate). The following precursor powders were utilized as starting materials: Bi_2_O_3_ (Alfa Aesar, Heysham, UK), K_2_CO_3_ (Fluka, Gillingham, UK), BaCO_3_ (Alfa Aesar, Heysham, UK), TiO_2_ (A-HR, Huntsman, Billingham, UK), CaCO_3_, (Sigma Aldrich, Gillingham, UK), and ZrO_2_ (E-101, Magnesium Elektron, Swinton, UK), all having >99% purity.

For the mixed ceramic, the raw starting powders were directly weighed and mixed according to the stoichiometry of the nominal composition. They were milled for 24 h in a polyethylene bottle using propan-2-ol as the suspension liquid and yttria-stabilized zirconia balls as the milling media, using a Megapot vibration mill (Pilamec Ltd., Lydney, UK). Then, the slurry was dried and calcined at 1300 °C for 4 h in a covered alumina crucible. The resulting powder was then mixed with 2 wt % extra Bi_2_O_3_ to compensate for volatilization losses during the subsequent sintering process. The as-calcined powder was milled for a further 72 h using the same process to break down any hard agglomerates and reduce the particle size. After drying, the resulting powder was mixed with 2 wt% polyethylene glycol solution (PEG 1500) as a lubricant and binder. Next, the samples were formed into pellets with a diameter of 10 mm and a thickness of around 1.5 mm by uniaxial pressing at a pressure of 150 MPa. The green pellets were sintered in two stages, which involved heating to 500 °C for 1 h to burn out the organic processing aids and then sintering at 1100 °C for 3 h, with heating and cooling rates of 5 °C min^−1^.

Alternatively, the separate preparation procedure involved the calcination of the KBT and BCZT powders individually for a period of 4 h at temperatures of 950 °C and 1300 °C, respectively. Afterward, the resulting KBT and BCZT powders were mixed in the appropriate ratio according to the formula of 0.65KBT-0.35BCZT, again using an addition of 2 wt% excess bismuth oxide. The mixture was then ball-milled for 72 h, followed by pressing and sintering at 1125 °C for 3 h, using the same heating and cooling rate of 5 °C min^−1^.

The Archimedes method was used to measure the bulk densities of the sintered samples, with water as the immersion medium. The dense sintered samples were lightly ground to obtain parallel and smooth faces. Some sintered samples of both types were annealed in an alumina crucible for 1 h at 1000 °C and then quenched in air by removing the crucible directly from the furnace and immediately placing it onto an alumina tile at room temperature. These are denoted ‘quenched’ (Q), while the as-sintered pellets are denoted ‘slow-cooled’ (SC).

X-ray diffraction (XRD) was employed for phase analysis at room temperature using an X’Pert-Pro diffractometer (Philips PANalytical, Almelo, The Netherlands) with CuKα radiation with a wavelength of 1.54060 Å, and Topas software version 5.0 was used to perform full-pattern Rietveld refinement. For the unpoled samples, the as-sintered ceramic pellets were crushed and ground into ceramic powders, followed by annealing at 550 °C for 30 min. For the determination of the crystal structure in the poled specimens, an air-dried silver paint was used to form the conductive electrodes prior to poling using the method described below. The electrodes were then removed using acetone. Scanning electron microscopy (Mira3 SC, Tescan-UK Ltd., Huntingdon, UK) was used to examine the microstructure of the polished cross-sections; in addition, the surfaces were chemically etched by immersion in an aqueous solution, containing 4% HCl and 1% HF for 20 s, to reveal the grain boundaries and ferroelectric domain configurations.

For the electrical measurements, disk-shaped samples were coated with silver paste (C2000107P3, Gwent Electronic Materials Ltd., Pontypool, UK) and annealed for 30 min at 550 °C to remove any organic binders. The measurements of the ferroelectric polarization electric field (P-E) hysteresis loops were performed using an HP33120A function generator (Hewlett-Packard, Palo Alto, CA, USA) coupled to an HVA1B Chevin Research HV amplifier (Chevin Research, Otley, UK) [37]. A burst-mode waveform comprising 4 cycles of a sinusoidal electric field at a frequency of 2 Hz was used, corresponding to a maximum electric field of 6 MV m^−1^. The induced current was measured using a current amplifier, where the current waveform was numerically integrated over time to yield charge. Subsequently, the polarization was computed as the surface charge density [37]. The same measurement procedure was used to provide AC-poled samples for subsequent electrical property measurements.

The temperature and frequency dependence of the low-field dielectric properties were determined by means of an automated measurement system comprising an LCR-meter (HP 4284A, Agilent Technologies, Amstelveen, The Netherlands,) connected to the electroded sample by conductive silver wires. The measurement temperatures were recorded with a thermocouple next to the alumina specimen holder inside of a CWF 1200 furnace (Carbolite-Gero Ltd., Hope Valley, UK). The relative dielectric permittivity, ε_r_, and the dielectric loss tangent, tan δ, were determined over a range of temperature (from 25 to 350 °C) and at different frequencies (1, 10, and 100 kHz) using a heating rate of 2 °C min^−1^.

The longitudinal strain–electric-field, *x_3_*–*E*, response was determined using a TF 2000 Ferroelectric Analyzer (AixACCT Systems GmbH, Aachen, Germany) using a triangular waveform at a frequency of 1 Hz. Quasi-static piezoelectric coefficients were measured for the poled samples using a d_33_ meter (APC International Ltd., Mill Hall, PA, USA). The thermally stimulated depolarization current (TSDC) measurements were performed using the method reported previously [15], yielding the depolarization temperature, *T_d_* [38].

## 3. Results and Discussion

### 3.1. Structural Characterization

**Figure 1:**
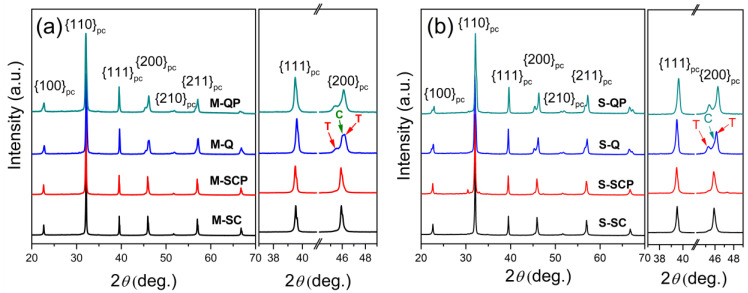
XRD patterns of 0.65KBT-0.35BCZT ceramics in various states: slow-cooled (SC), slow-cooled-poled (SCP), quenched (Q), and quenched-poled (QP) for (**a**) mixed (M) and (**b**) separate (S) calcination procedures.

The crystal structure of the 0.65KBT-0.35BCZT ceramic prepared according to the separate calcination procedure was discussed in depth using the synchrotron X-ray powder diffraction results reported in our previous investigation, which identified the presence of coexisting tetragonal and cubic phases [15]. The XRD data obtained using a laboratory diffractometer in this study were employed to evaluate the differences between the mixed and separately calcined ceramics at room temperature, as shown in Figure 1. It is evident that both types exhibited a perovskite structure, yielding similar diffraction peak profiles. The constituent phases (cubic and tetragonal) were also the same, but there were significant differences in the phase fractions. The most obvious effect was an increase in the tetragonal (T) phase content in quenched, *Q*, and quenched-poled, *QP*, samples, indicated by the splitting of the (002) and (200) peaks in the region of 46° 2θ. In contrast, the slow-cooled, *SC*, and slow-cooled-poled, *SCP*, materials appear to be predominantly cubic (C), although a low-angle shoulder on the {200}_pc_ (pseudo-cubic structure) peak profile of these samples suggests the presence of a coexisting tetragonal phase.

**Figure 2:**
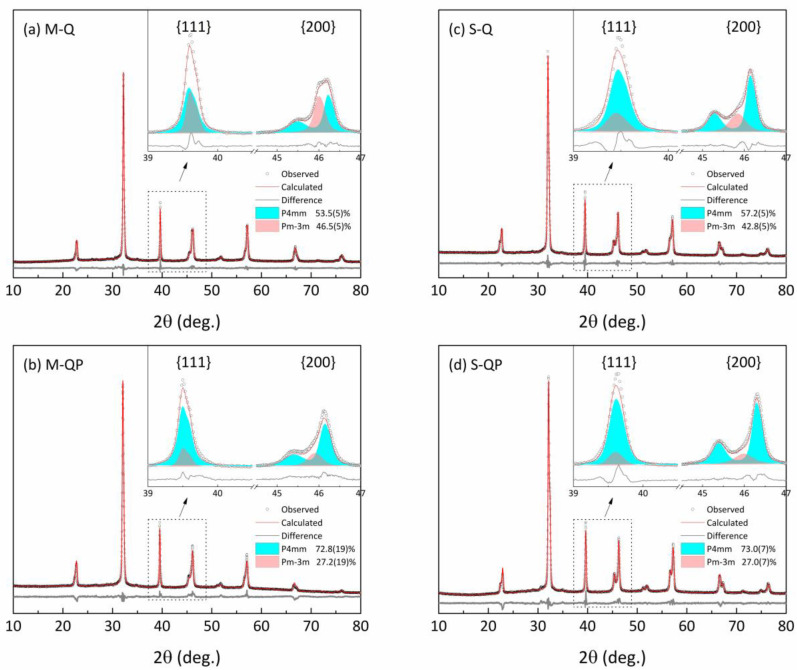
Experimental, calculated, and difference data obtained by full-pattern refinement of XRD patterns showing {111}_pc_ and {200}_pc_ peak profiles of KBT-BCZT ceramics in various states: (**a**) quenched (*Q*), (**b**) quenched-poled (*QP*) for mixed (*M*) calcination, (**c**) quenched (*Q*), and (**d**) quenched-poled (*QP*) for separate (*S*) calcination. Arrows indicate the selected peak profiles shown in the inset figures.

Full-pattern refinement was subsequently performed for both the *Q* and *QP* powders, as shown by the results presented in Figure 2 and Table 1. For the quenched ceramics, it was found that that the *M* sample, Figure 2a, contained a greater cubic phase fraction of 46.5% in comparison with 42.8% for the *S* specimen, Figure 2c, leading to relaxor ferroelectric behavior in both cases. Furthermore, the application of an electric field for the poling process resulted in clear splitting of the {200}_pc_ tetragonal peak profile and enhanced tetragonal phase fraction in both cases, as illustrated in Figure 2b,d. These structural modifications also gave rise to significant changes in the microstructure and the dielectric and ferroelectric properties, as reported in the following sections.

### 3.2. Microstructure

**Figure 3:**
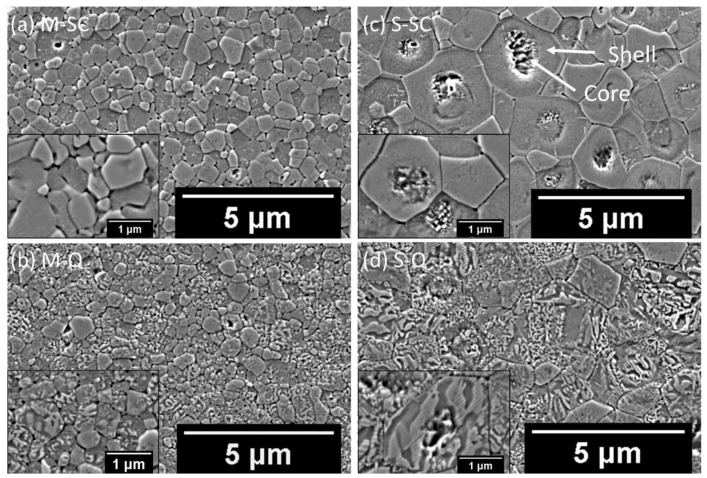
Backscattered electron (BSE) images of chemically etched surfaces of 0.65KBT-0.35BCZT ceramics in various states: slow-cooled *(SC*) and quenched (*Q*) for (**a**,**b**) mixed (*M*), (**c**,**d**) separate (*S*) calcination procedures.

Following sintering, both *M-* and *S-*type ceramics exhibited high values of absolute density, at around 5.58 g cm^−3^. This level exceeds 92% of the theoretical density of 6.04 g cm^−3^, calculated from the results of full-pattern refinement of the XRD patterns using Topas software version 5.0 [39]. The polished cross-sections of the mixed and separately calcined ceramics, for both slow-cooled and quenched states, were examined in order to study the effect of the reaction pathway on the microstructure. The SEM images of the chemically etched surfaces are presented in Figure 3. The results demonstrate that chemical heterogeneity in the form of core-shell-type features is present in the separately calcined samples, as illustrated in Figure 3c,d. In our previous study, we interpreted such a microstructure in terms of pseudo-cubic relaxor ferroelectric KBT-rich shell regions and tetragonal ferroelectric BCZT-rich cores [13].

In contrast, the mixed calcination ceramics exhibited a homogenous microstructure, devoid of any core-shell features, as illustrated in Figure 3a,b. Wide grain size distributions were observed in both cases. However, the mixed ceramic exhibited a generally smaller grain size, in a range from 0.5 to 1 μm, while that of the separately calcined material was in a range from 1 to 3 μm, as shown in Figure 3c,d, respectively. The quenched samples, shown in Figure 3b,d, exhibit distinct and uniform ferroelectric domain configurations throughout the grains.

**Figure 4:**
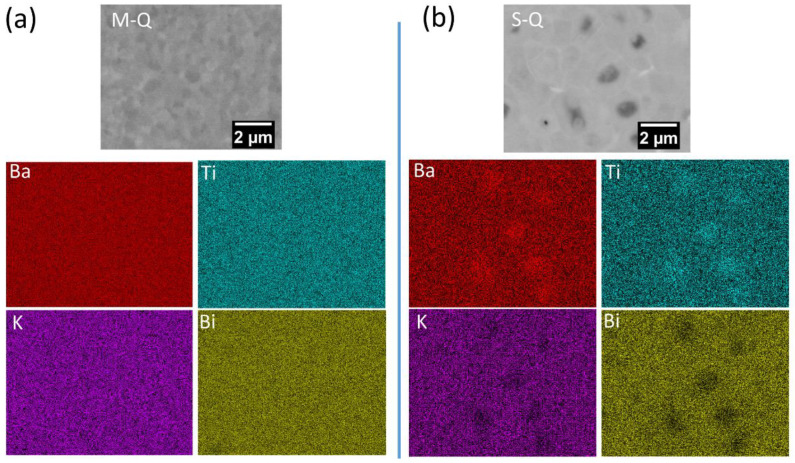
SEM-EDX elemental mapping results for (**a**) M- and (**b**) S-type 0.65KBT-0.35BCZT ceramics, showing homogeneous and core-shell-type microstructures, respectively.

Regions of darker contrast, corresponding to a lower average atomic number, were evident in the grain centers of the S ceramic, but not in the case of the more homogeneous M material, as illustrated by the back-scattered electron images shown in Figure 4. The corresponding elemental maps exhibited more intense Ba and Ti concentrations in the darker regions of the SEM image, associated with the grain cores, whereas Bi and K were slightly enriched in the surrounding brighter areas. These findings indicate that the use of separately calcined KBT and BCZT components favors the formation of BCZT-rich core and KBT-rich shell regions. We suppose that the BCZT solid solution forms relatively unreactive large particles, which act as nuclei (core regions) during sintering with KBT that consolidates around these cores to form the surrounding shell due to its greater reactivity at low temperatures, forming a metastable heterogeneous microstructure [17,34].

On the other hand, a relatively homogenous microstructure was observed for the mixed calcination ceramic, with no obvious evidence of chemical heterogeneity. In the related Bi-based solid solution, Bi_0.5_Na_0.5_TiO_3_–25SrTiO_3_ (BNT-25ST), it was reported that the use of a one-step calcination procedure led to considerable compositional enrichment of BNT in the grain cores. This was attributed to the different reactivities of the two components, with BNT being produced initially to form the core and ST at a later stage to create the shell regions [17,35]. However, it was also reported that the specific processing parameters used to prepare BNT-25ST ceramics have an important influence on micro-chemical homogenization, with a reduction in the core density (from ~0.12 to ~0.08 cores/μm^2^) as the calcination temperature increased from 1000 to 1150 °C [34]. In the present case, it is apparent that the calcination and sintering temperatures employed for one-step calcination of the mixed KBT-BCZT ceramics were sufficiently high enough to enable the homogenization of each element throughout the grains. The relative ease of homogenization for KBT-BCZT solid solutions could be associated with the formation of a low melting point, transient liquid phase between K_2_O and Bi_2_O_3_, provided that the other components exhibit reasonably high solubility in such a liquid.

### 3.3. Dielectric and Ferroelectric Properties

**Figure 5:**
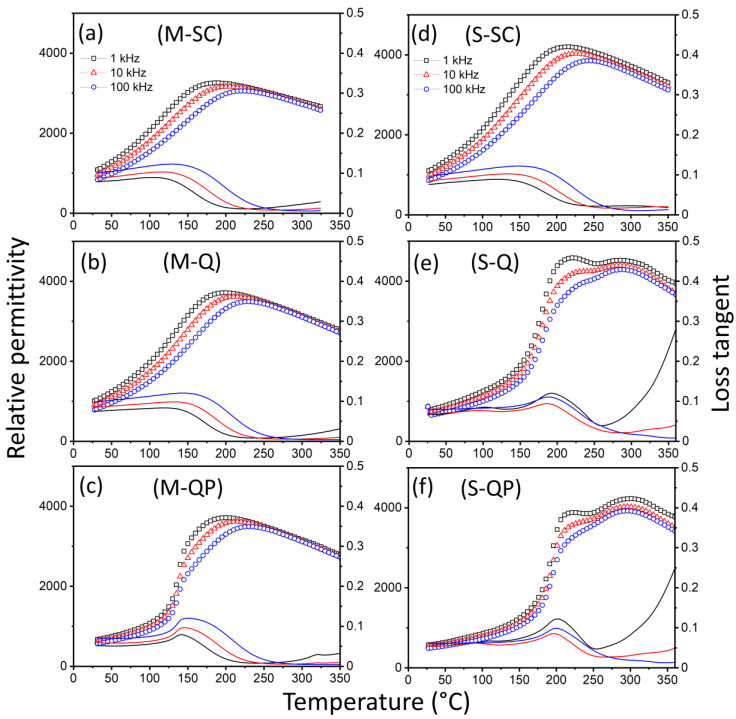
Temperature dependence of relative permittivity and loss tangent of 0.65KBT-0.35BCZT ceramics in various states: slow-cooled (*SC*), quenched (*Q*), and quenched-poled (*QP*) for (**a**–**c**) mixed (*M*) and (**d**–**f**) separate (*S*) calcination procedures. Individual data points represent the relative permittivity values while solid lines show the dielectric loss.

The ε_r_–T and tan δ–T relationships for the KBT-BCZT ceramics are presented in Figure 5. The relaxor ferroelectric nature of the materials is evident in the frequency-dependent reduction in ε_r_ at temperatures below those associated with the peak permittivity value, T_m_, and the increase in T_m_ with increasing frequency. The slow-cooled specimens exhibit one broad peak in ε_r_, with T_m_ ~225 °C and ~250 °C (at 100 kHz) for the *M-SC-* and *S-SC*-type materials, respectively, as illustrated in Figure 5a,d. Although generally similar, the behavior of the *M* sample is more diffuse and dispersive than that of the *S*-type. The application of the quenching process revealed further differences between the materials, with two distinct dielectric anomalies being evident for the heterogenous core-shell-type *S* specimen and only a single broad peak for the more homogeneous *M* sample, as shown by the results presented in Figure 5b,e. These observations support the argument that the two peaks in the *S* sample can be attributed to a combination of contributions from the shell and core regions, as proposed previously [15].

The subsequent poling of the quenched samples yielded further differences between the stability of the ferroelectric states in the *M* and *S* samples, illustrated by the ε_r_–T and tanδ–T relationships presented in Figure 5c,f. For the *M* sample, a steepening and reduced frequency dependence of the *ε_r_*–*T* curve was identified in the region of 135 °C, which is associated with the depolarization temperature reported below. In contrast, the poling process had a relatively minor effect on the ε_r_–T curves for the *S* sample, yielding only a slight increase in the temperature associated with the steep part of the curve, from ~175 to ~190 °C.

The diffuse phase transition (DPT) behavior of the KBT-BCZT ceramics was evaluated further in terms of the modified Curie–Weiss Law, represented by Equation (1), and the associated diffuseness parameter, γ, shown by the plots presented in Figure S1. The γ values obtained for the homogeneous *M* materials were 1.56, 1.58, and 1.59 for M-SC, M-Q, and M-QP, respectively; moreover, these are remarkably similar in comparison with the significant variations in ferroelectric properties as a function of the heat treatment and poling procedures, reported below. Nevertheless, the γ values are broadly consistent with the non-ergodic relaxor ferroelectric characteristics of the materials.

(1) $$\frac{1}{\varepsilon_{r}}-\frac{1}{\varepsilon_{m}}=\frac{{\left(T-T_{m}\right)}^{\gamma }}{C}$$

The dielectric losses of the various types of KBT-BCZT ceramics are similar in the low temperature range, below 200 °C, where the dominant extrinsic loss mechanisms are associated with ferroelectric domain walls and/or polar nanoregions. On the other hand, the dielectric losses of the *S-Q* and *S-QP* samples at high temperature/low frequencies are significantly higher than those in the slow-cooled state, indicating a higher contribution from the conduction losses after quenching. These observations are consistent with a thermally activated conduction process governed by n-type electronic conductivity due to partial reduction at high temperatures, according to the following defect chemical equation:

(2) $$O_{O}^{x}\to V_{O}^{⦁⦁}+2e^{\prime }+\frac{1}{2}O_{2}(g)$$

According to this interpretation, the higher concentrations of the conducting species are retained by quenching, while they are reduced by re-oxidation during slow cooling. On this basis, annealing in the air at intermediate temperatures (~500 °C) could potentially be employed as a method to reduce the conduction losses in the quenched samples while retaining their enhanced ferroelectric properties. The relative insensitivity of such losses to the quenching process in the more homogeneous, mixed calcination ceramics is attributed to the stabilization of the 0.65KBT-0.35BCZT solid solution by the refractory oxides, BaO, CaO, and ZrO_2_, leading to a reduction in the oxygen vacancy concentration at high temperatures.

**Figure 6:**
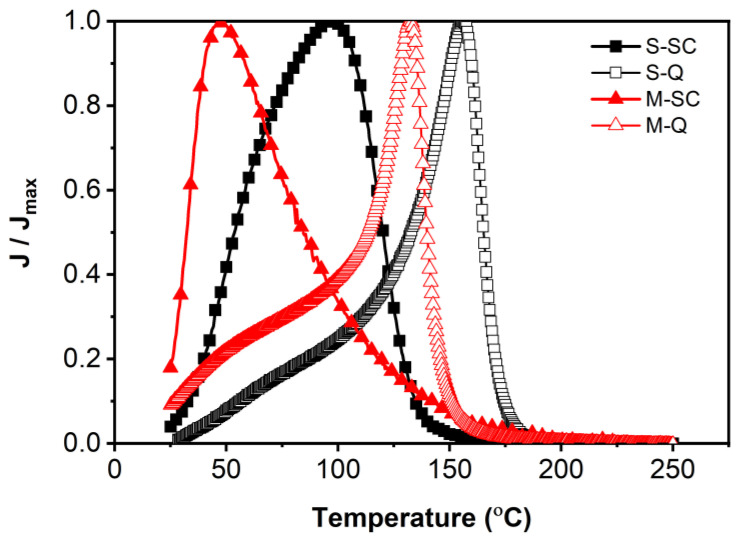
Thermal depolarization results for 0.65KBT-0.35BCZT ceramics prepared using mixed (*M*) and separate (*S*) calcination procedures in slow-cooled (*SC*) and quenched (*Q*) states, presented in terms of the normalized current density, J/J_max_.

The depolarization temperature, T_d_, for 0.65KBT-0.35BCZT ceramics prepared under different processing and heat treatment conditions was identified as the temperature corresponding to the peak in the current density–temperature, J–T, relationship [38], as shown in Figure 6. Prior to measurement, the samples were poled at room temperature in a silicone oil bath by applying several cycles of an AC field with an amplitude of 6.0 MV m^−1^ until stable remanent polarization was obtained. The use of quenching increased the T_d_ values of each specimen type from ~100 to ~160 °C for the *S* sample, and from ~50 to ~130 °C for the *M* sample. A broad shoulder is evident in the J–T curve for the *S-SC* sample, with an inflection at ~60 °C, as illustrated in Figure 6. However, this was largely suppressed after quenching, with a slight increase in the temperature of the inflection to ~75 °C. These observations are consistent with those of the dielectric permittivity in terms of depolarization temperature.

### 3.4. Thermal Evolution of Ferroelectric and Electromechanical Properties

**Figure 7:**
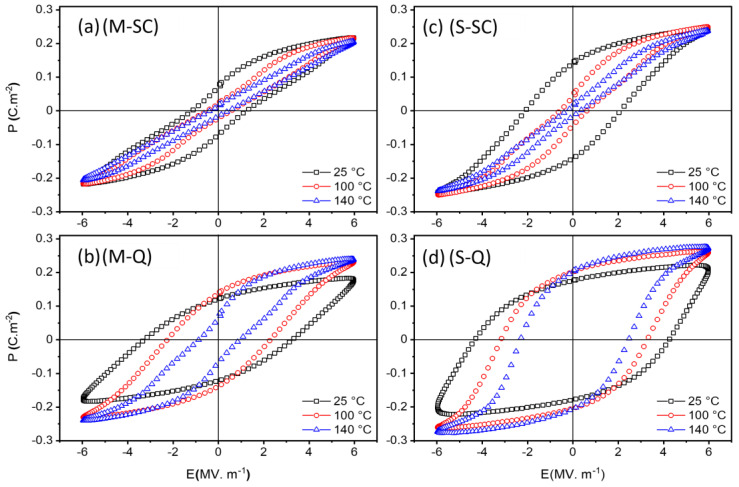
P-E hysteresis loops of slow-cooled (*SC*) and quenched (*Q*) 0.65KBT-0.35BCZT ceramics prepared using (**a**,**b**) mixed (*M*) and (**c**,**d**) separate (*S*) calcination procedures.

**Figure 8:**
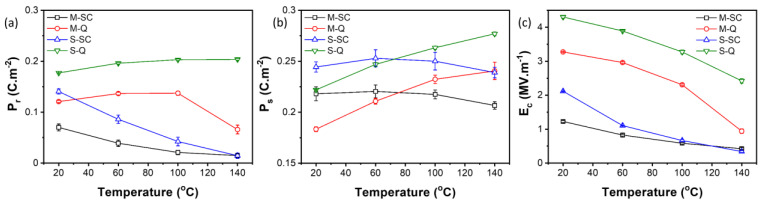
Variations in (**a**) *P_r_*, (**b**) *P_s_*, and (**c**) *E_c_* as a function of temperature for 0.65KBT-0.35BCZT ceramics with different processing procedures: parameters were determined from the ferroelectric *P-E* hysteresis loops.

Representative ferroelectric hysteresis loops and the associated parameters P_r_, P_s_, and E_c_ at specific temperatures for the slow-cooled and quenched KBT-BCZT ceramics are shown in Figure 7 and Figure 8, respectively. At room temperature, the slow-cooled materials for both types of calcination exhibited irreversible ferroelectric domain switching characteristics, as shown in Figure 7a,c, with the separately calcined core-shell-type sample displaying significantly higher polarization and coercive field values. The P-E loops for both types of material became progressively constricted during heating, characteristic of the nanopolar, ergodic relaxor ferroelectric state and its reversible polarization switching behavior. Thermally induced disruption of the ferroelectric order is also indicated by the reduction in negative electrostrain on heating, as discussed further below. The *S*-type ceramic exhibited consistently higher P_r_, P_s_, and E_c_ values than those of the *M*-type material, but with similar temperature dependence, indicating a higher stability of the ferroelectric ordering.

After quenching, the thermally induced constriction of the P-E loop for the mixed calcination sample became evident only at temperatures above 100 °C, yielding a significant reduction in P_r_ at 140 °C, as shown in Figure 7b. In contrast, for the separately calcined case, Figure 7d, both the remanent and saturation polarization values increased upon heating as a result of the gradual reduction in coercive field and the enhancement of domain switching behavior. According to the microstructural observations shown in Figure 3 and Figure 4, the *S-SC* ceramic is a composite-type material comprising a BCZT-rich ferroelectric core and KBT-rich relaxor ferroelectric shell regions within the grains. For the shell regions, we suppose that both non-ergodic and ergodic PNRs co-exist, with the proportion of non-ergodic regions reducing gradually upon heating due to the disruption of the ferroelectric order. The application of the quenching process stabilized the non-ergodic PNRs within the shell regions, leading to more open P-E loops and improved ferroelectric switching characteristics upon heating.

The mixed calcination method led to the formation of a more homogeneous ceramic, without any evidence of core-shell-type microstructural heterogeneity. The *M-SC* sample has a dominant ergodic relaxor ferroelectric response at room temperature, with the small but non-zero remanent polarization suggesting the presence of some non-ergodic PNRs. Therefore, the P-E loops are generally more constricted than those of the *S-SC* ceramic due to the absence of a ferroelectric core. The application of the quenching process led to significant improvements in ferroelectric switching behavior, but with reduced stability at temperatures greater than 100 °C, as indicated by the reduction in *P_r_*.

**Figure 9:**
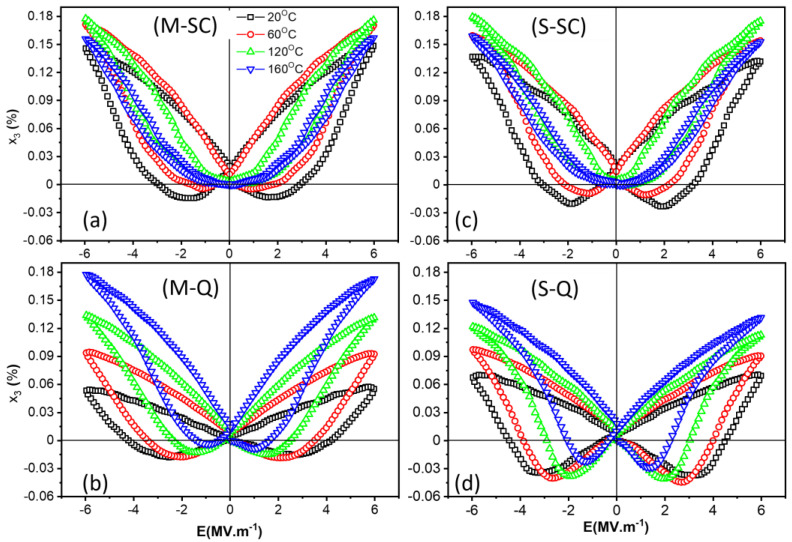
Temperature dependence of longitudinal strain–electric-field loops for slow-cooled and quenched 0.65KBT-0.35BCZT ceramics prepared using (**a**,**b**) mixed (*M*) and (**c**,**d**) separate (*S*) calcination procedures.

**Figure 10:**
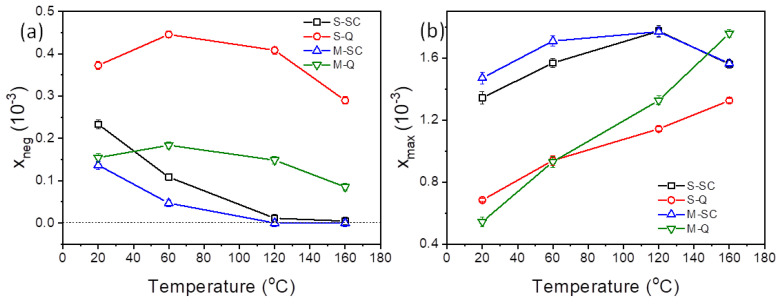
Temperature dependence of (**a**) negative strain and (**b**) maximum strain parameters for slow-cooled (*SC*) and quenched (*Q*) 0.65KBT-0.35BCZT ceramics.

Bipolar longitudinal strain–electric-field, x_3_–E, hysteresis loops of the 0.65KBT-0.35BCZT ceramics, measured at temperatures in the range 20 to 160 °C, are presented in Figure 9. The thermal evolution of the maximum and negative strain parameters derived from these hysteresis loops is displayed in Figure 10. The x_3_–E relationships show the same general trends as those observed in the P-E loops and are consistent with our interpretation of the ferroelectric responses of the *S* (core-shell) and *M* (homogeneous) ceramics.

The emergence of ‘sprout-shaped’ (unipolar) x_3_–E loops with reduced hysteresis upon heating in the slow-cooled materials confirms the development of the reversible ergodic relaxor ferroelectric response. Conversely, the presence of a negative strain, x_neg_, around the coercive field for the quenched samples shows evidence of 90° domain switching and indicates irreversible non-ergodic relaxor ferroelectric behavior. The enhancement of domain switching during heating is obvious in both types of quenched samples, as indicated by reductions in the coercive field and the progressive increase in the maximum strain values. However, a slight decrease in the negative strain is evident at temperatures above 60 °C. For the *M-Q* sample, this reduction in x_neg_ is associated with the gradual constriction of the *P-E* loops shown in Figure 7b and indicates an increasing tendency for reversible polarization switching behavior. The *S-Q* sample exhibited a more stable ferroelectric response during heating, which is corroborated by the significantly higher values of the negative strain, as shown in Figure 10a.

**Figure 11:**
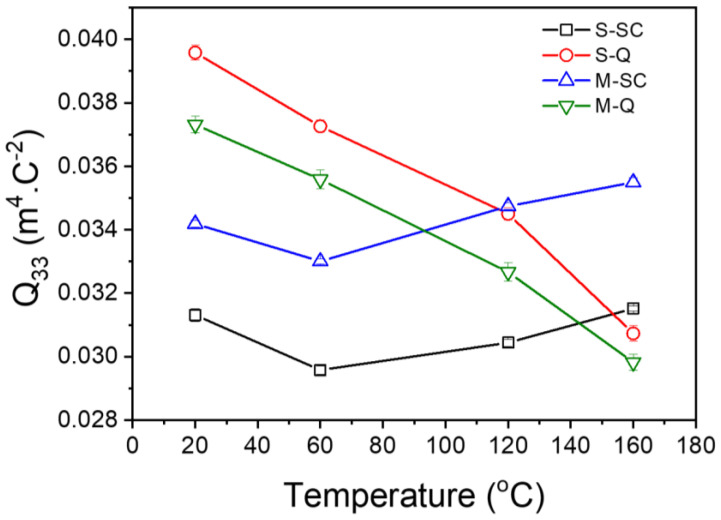
Temperature dependence of electrostrictive coefficient, *Q*_33_, of 0.65KBT-0.35BCZT ceramics.

The P-E and x_3_–E relationships of the KBT-BCZT ceramics were replotted as x_3_–P and x_3_–P^2^, as shown in Figures S2 and S3, respectively (Supplementary Materials). Subsequently, the data extracted from the x_3_–P^2^ relationships were used to determine the electrostrictive coefficient, Q_33_, as illustrated in Figure 11. Both of the quenched samples exhibited progressive reduction in Q_33_ during heating, with the *S-Q* specimen exhibiting consistently higher values throughout the temperature range. In contrast, the slow-cooled samples both exhibited a slight reduction in Q_33_ up to 60 °C, followed by a gradual increase upon further heating. However, a higher value at room temperature was observed for the *M-SC* ceramic. The derived values of the piezoelectric and electrostrictive coefficients are summarized in Table 2.

## 4. Conclusions

The results of this study demonstrate that the choice of suitable solid-state reaction processing procedures and heat treatment parameters can be used to control the microstructure and ferroelectric properties of 0.65KBT-0.35BCZT ceramics. The synthesis of KBT and BCZT components separately (*S*), prior to sintering, led to the formation of a relatively large-grained heterogeneous microstructure comprising BCZT-rich core and KBT-rich shell regions with tetragonal ferroelectric and pseudo-cubic relaxor ferroelectric characteristics, respectively. Conversely, a homogenous solid solution with relaxor ferroelectric properties and a pseudo-cubic structure was produced by the mixed (*M*) calcination procedure.

It was observed that the application of quenching enhances ferroelectric ordering, either across the grains for the *M*-type KBT-BCZT ceramic or in the shell regions for the *S*-type material. This effect is interpreted in terms of the preservation of the more chemically homogenous high temperature state within each of these regions by quenching. However, the field-induced ferroelectric state transformed back to relaxor-like behavior during heating, in accordance with a gradual structural transformation from tetragonal to pseudo-cubic structures. These findings are supported by the results of the temperature-dependent dielectric and ferroelectric measurements. The application of the quenching process led to an increase in the depolarization temperature from ~100 to ~160 °C for the *S* sample, and from ~50 to ~130 °C for the *M* sample. This work demonstrates that the microstructure in KBT-BCZT ceramics can be strategically engineered to control the electromechanical response by the suitable modification of the processing procedures; in addition, a similar approach could also be explored in other complex solid-solution systems.

## Figures and Tables

**Table 1:** Structural parameters of KBT-BCZT ceramics in the quenched (*Q*) and quenched-poled (*QP*) states for the mixed (*M*) and separately (*S*) calcined ceramics, at room temperature. GoF denotes the goodness of fit.

Sample Type	Phase Fraction (%)	Lattice Parameter		(c/a)_T_	GoF
		a (Å)	c (Å)		
*M-Q*	T = 53.5(5) C = 46.5(5)	a_T_ = 3.95331(14) a_C_ = 3.97152(12)	c_T_ = 4.0154(3)	1.0157	1.88
*M-QP*	T = 72.8(19) C = 27.2(19)	a_T_ = 3.9557(3) a_C_ = 3.9758(3)	c_T_ = 4.0182(5)	1.0158	2.31
*S-Q*	T = 57.2(5) C = 42.8(5)	a_T_ = 3.94962(12) a_C_ = 3.97613(19)	c_T_ = 4.02212(19)	1.0185	1.96
*S-QP*	T = 73.0(7) C = 27.0(7)	a_T_ = 3.95016(15) a_C_ = 3.9767(3)	c_T_ = 4.0266(2)	1.0194	2.25

**Table 2:** Summary of room temperature piezoelectric and electrostrictive coefficients for 0.65KBT-0.35BCZT ceramics. The values of d_33_* represent the effective high-field piezoelectric strain coefficient, given by the ratio x_max_/E_max_ at 6 MV m^−1^.

	*S*		*M*	
	SC	Q	SC	Q
d_33_ (pC N^−1^)	40	82	16	71
d_33_* (pm V^−1^), x_max_/E_max_	240	113	246	91
Q_33_ (m^4^ C^−2^)	0.031	0.04	0.034	0.037

## Data Availability

The data that support the findings of this study are available from the corresponding author upon reasonable request due to privacy.

## Supplementary Materials

The following supporting information can be downloaded at: https://www.mdpi.com/article/10.3390/ma18030623/s1, Figure S1: Logarithmic plots of dielectric data for 0.65KBT-0.35BCZT ceramics at temperatures above T_m_, used for determination of diffuseness factor, γ. Figure S2: Axial strain-polarisation (x_3_-P) relationships for slow cooled (*SC*) and quenched (*Q*) KBT-BCZT ceramics prepared using (a-b) mixed (*M*) and (c-d) separate (*S*) calcination procedures, measured at temperatures from 20 to 160 °C. Figure S3: x_3_-P^2^ relationships for slow cooled (*SC*) and quenched (*Q*) KBT-BCZT ceramics prepared using (a-b) mixed (*M*) and (c-d) separate (*S*) calcination procedures, measured at temperatures from 20 to 160 °C.
