# Supplementary material for: Microstructural Engineering of Ferroelectric and Electromechanical Properties in 0.65KBT-0.35BCZT Ceramics
Source: Materials (Basel). 2025 Jan 29;18(3):623. doi: 10.3390/ma18030623 (PMC11819832; doi:10.3390/ma18030623)
Supplement: Supplementary file 1 [file materials-18-00623-s001.zip › materials-3341316-SI.pdf]

Microstructural engineering of ferroelectric and electromechanical properties in 0.65KBT-0.35BCZT ceramics.

Mohammed N. Al-Aaraji [1,2*], Bing Wang [3,4], Antonio Feteira [5] and David A. Hall [2,*]

[1] Department of Ceramic Engineering and Building Materials, College of Materials Engineering, University of Babylon, Al Hilla, Iraq.

[2] Department of Materials, University of Manchester, Manchester, M13 9PL, UK.

[3] College of Civil and Transportation Engineering, Shenzhen University, Shenzhen 518060, China.

[4] Institute for Advanced Study, Shenzhen University, Shenzhen 518060, China.

[5] Materials and Engineering Research Institute, Sheffield Hallam University, Sheffield, S1 1WB, UK.

*Correspondence: mohammed.al_aaraji@uobabylon.edu.iq and david.a.hall@manchester.ac.uk

Supplementary Materials

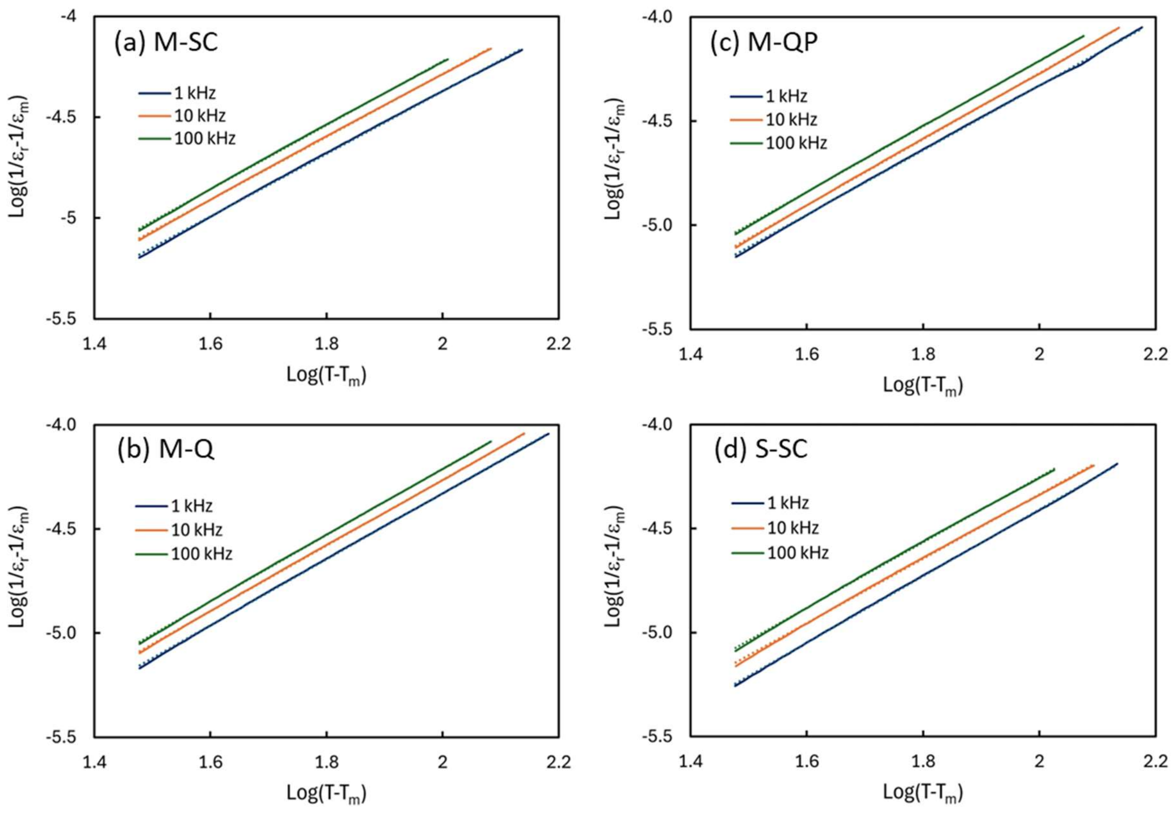

Figure S1. Logarithmic plots of dielectric data for 0.65KBT-0.35BCZT ceramics at temperatures above T_m, used for determination of diffuseness factor, γ. Solid lines show the experimental data while the dashed lines represent the linear fit according to equation 1.

$$Log\left(\frac{1}{\varepsilon_r} - \frac{1}{\varepsilon_m}\right) = \gamma\, Log(T - T_m) - Log(C) \quad (1)$$

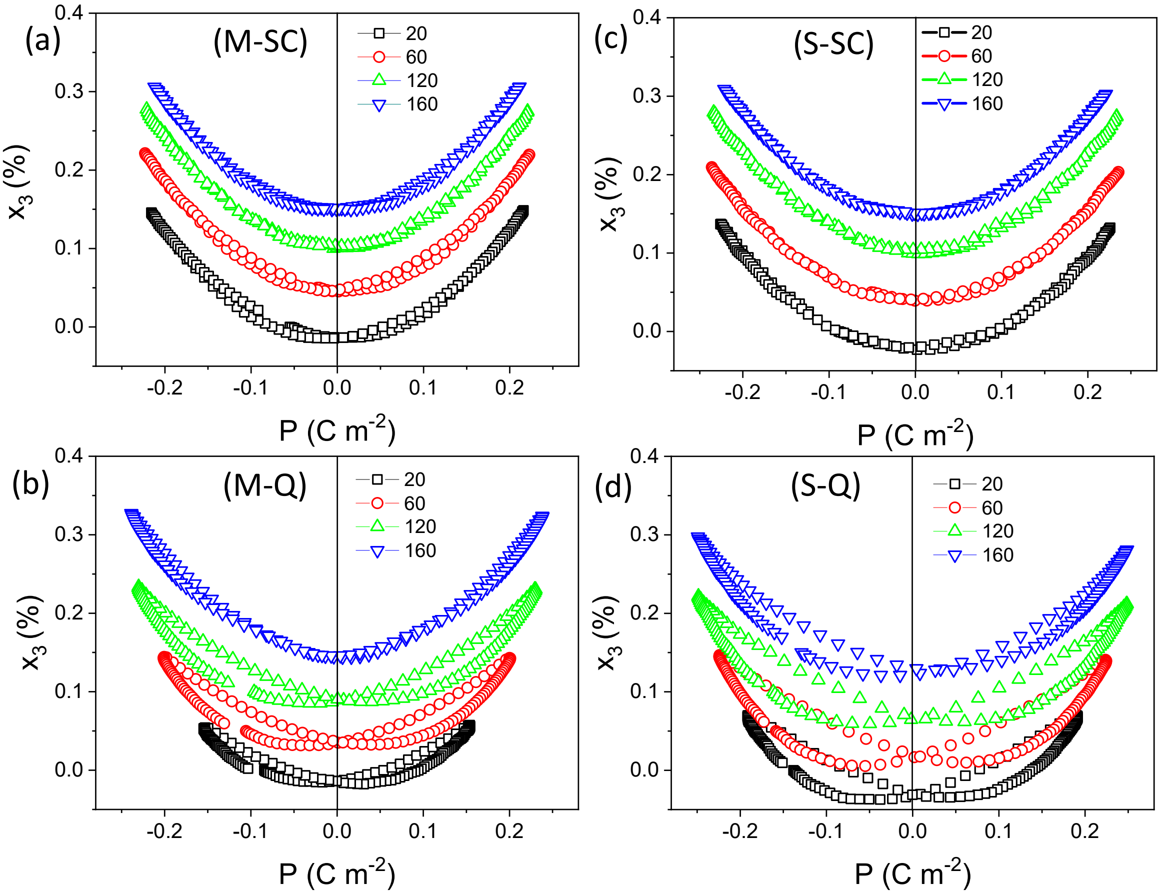

Figure S2. Axial strain-polarisation (*x_3-P)* relationships for slow cooled (*SC*) and quenched (*Q*) KBT-BCZT ceramics prepared using (a-b) mixed (*M*) and (c-d) separate (*S*) calcination procedures, measured at temperatures from 20 to 160 °C. Note that the x_3 values are offset vertically by an arbitrary amount for clarity.

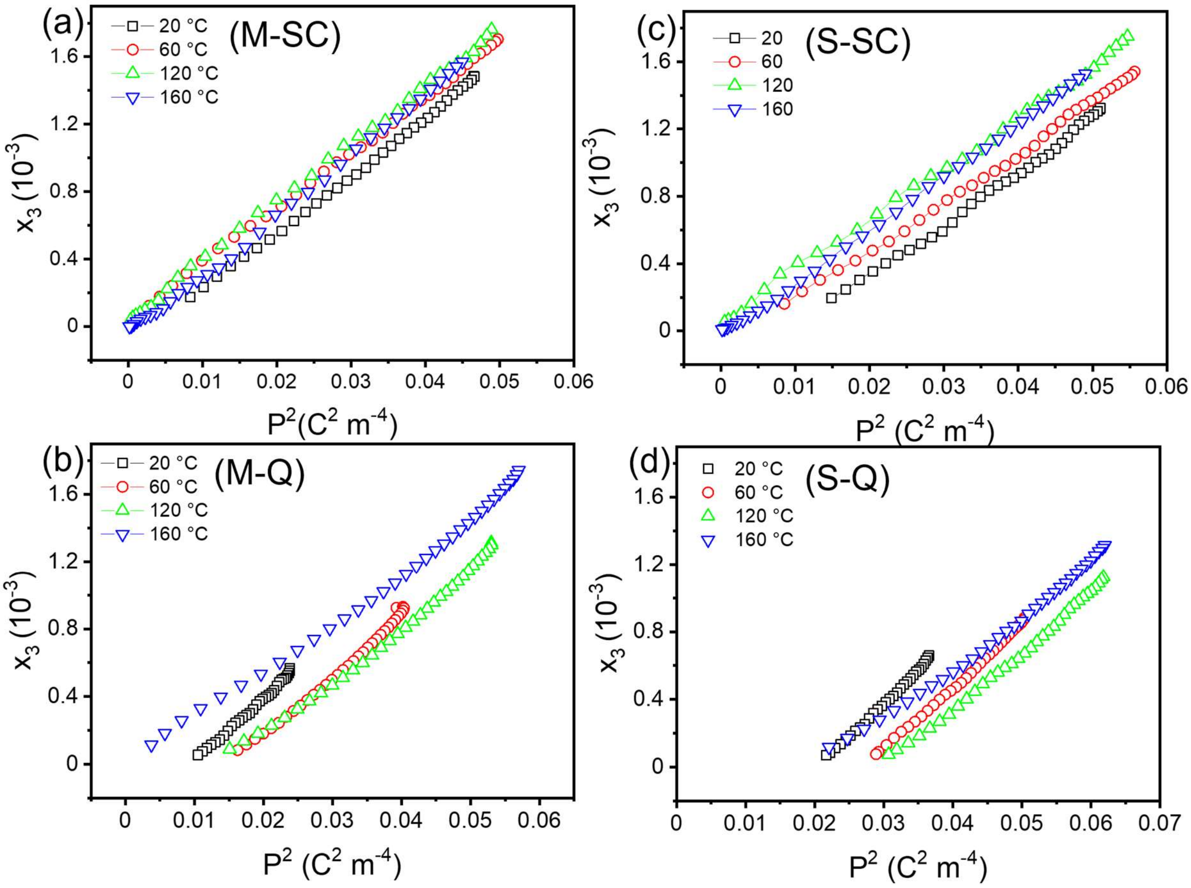

Figure S3. x_3-P^2 relationships for slow cooled (*SC*) and quenched (*Q*) KBT-BCZT ceramics prepared using (a-b) mixed (*M*) and (c-d) separate (*S*) calcination procedures, measured at temperatures from 20 to 160 °C.
